# “GONE WITH THE WIND”: The Transitory Effects of COVID-19 on the Gynecological System

**DOI:** 10.3390/jpm13020312

**Published:** 2023-02-10

**Authors:** Miriam Dellino, Antonella Vimercati, Antonio D’Amato, Gianluca Raffaello Damiani, Antonio Simone Laganà, Ettore Cicinelli, Vincenzo Pinto, Antonio Malvasi, Salvatore Scacco, Andrea Ballini, Leonardo Resta, Giuseppe Ingravallo, Eugenio Maiorano, Gerardo Cazzato, Eliano Cascardi

**Affiliations:** 1 Department of Biomedical Sciences and Human Oncology, University of Bari, 70121 Bari, Italy; 2 Department of Precision and Regenerative Medicine and Jonic Area, University of Bari “Aldo Moro”, 70121 Bari, Italy; 3 Unit of Gynecologic Oncology, ARNAS “Civico—Di Cristina—Benfratelli”, Department of Health Promotion, Mother and Child Care, Internal Medicine and Medical Specialties (PROMISE), University of Palermo, 90127 Palermo, Italy; 4 Department of Basic Medical Sciences and Neurosciences, University of Bari “Aldo Moro”, 70121 Bari, Italy; 5 Department of Precision Medicine, University of Campania “Luigi Vanvitelli”, 80138 Naples, Italy; 6 Department of Medical Sciences, University of Turin, 10124 Turin, Italy; 7 Pathology Unit, FPO-IRCCS Candiolo Cancer Institute, 10060 Candiolo, Italy

**Keywords:** COVID-19, SARS-CoV-2, vaccine, gynecological and obstetric effects, anomalous uterine bleeding

## Abstract

The coronavirus disease no longer seems to represent an insurmountable global problem. This is thanks to the advent of coronavirus vaccines, which have alleviated the most serious symptoms associated with this disease. On the other hand, there are still many extrapulmonary symptoms of COVID-19, and among these also those of a gynecological nature. At the moment, there are several questions in this field, one above all concerns the causal link between COVID-19, vaccines and gynecological alterations. Furthermore, another important aspect is represented by the clinical impact of post-COVID-19 gynecological alterations on the female population which, to date, would seem to be mainly due to their duration, even if the extent of these symptoms is still poorly understood. Furthermore, it is not possible to foresee eventual long-term aggravations, or more serious symptoms caused by other viral variants that may arrive in the future. In this review, we focus on this theme and attempt to reorganize the different pieces of a puzzle which, to date, does not seem to have shown us its complete picture.

## 1. Introduction

The factors that can induce alterations or gynecological pathologies within our organism are various and numerous. Most of these, such as hormonal imbalances, weight loss and stress, have been extensively investigated and we are now well aware of their symptoms, causes and effects [1,2]. Conversely, others are less clear. This can be due to a lack of in-depth knowledge of a problem but also to the fact that very often new contributing causes are identified that can manifest similar symptoms [3]. In this regard, coronavirus 2019 (COVID-19) is certainly an emerging pathology, many of whose symptoms and effects remain to date not widely clarified. The main symptoms of coronavirus COVID-19 are of a respiratory nature (fever, cough and dyspnea, acute respiratory syndrome) [4,5], due to its ability to act on pneumocytes II [6] through the angiotensin-2 receptor (ACE2). However, other complications are also known that can affect the sense of smell [7], sight (eye pain, redness and conjunctivitis) and the cardiovascular system [8,9,10] as well as other districts [11,12,13,14,15,16,17,18,19,20,21], highlighting how this pathology is now to be considered multiorgan.

The advent of anti-COVID-19 vaccines [22] has made this pathology more manageable globally, considering that their main effect was to ease the severity of symptoms as well as to reduce contagion. Generally, the most frequent side effects of COVID-19 vaccination are myalgia, pain in the inoculation arm, fever and asthenia [23]. Regarding alterations in the menstrual cycle or in the female reproductive system, currently there are no robust scientific data regarding adverse effects after vaccination. However, in clinical experience, following the administration of all COVID-19 vaccines, both mRNA and adenoviruses vectorized adenoviruses, some adverse drug reactions of the menstrual cycle have been recorded [12].

Furthermore, these disorders could have a high incidence in the general population but the evaluation of the causal link with the administration of the vaccination is not immediate except as a temporal consequence [24].

To date, this topic remains widely debated both by specialized health professionals who are collecting numerous reports relating to disorders of the menstrual cycle following the vaccine [12,14,25], as well as in global studies that are investigating the question through case–control analysis with an unvaccinated population [24]. Moreover, the extent of this problem is demonstrated by the global interest of the media and of women who have experienced menstrual changes, including altered menstrual duration, frequency, regularity and volume (heavier bleeding and clotting), as well as increased dysmenorrhea and worsening of premenstrual syndrome [25].

In this article, we aim to perform an evaluation of the current scientific literature documenting changes in the characteristics of the menstrual cycle during the COVID-19 pandemic, and provide suggestions for future research. We believe that we have a duty to understand the etiology and extent of this phenomenon in order to provide informed and reassuring answers to patients [26]. Furthermore, questions about menstruation have been ruled out by most large-scale COVID-19 studies (including vaccine trials), so we do not currently have clear information about the number of women who have experienced changes in their menstrual cycle, the duration of these changes, whether they reflect normal, predictable fluctuations in menstrual characteristics over time or rather are impacted by an exposure (e.g., pandemic restrictions, infection/disease, treatment, vaccine) or the precise nature of this exposure [12,27].

PubMed, Scopus and prepress servers (BioRxiv and MedRxiv) were searched in all fields for menstruation-related terms and COVID-19. We identified seven studies that report the characteristics of the menstrual cycle in relation to the virus in the period of the pandemic, and three on the gynecological impact of COVID-19 vaccines (Table 1 and Table 2).

## 2. Premenopausal Menstrual Cycle Alterations Not COVID-19/VACCINE-Related

The majority of the world’s population can attest to how menstrual disorders are very common and also debilitating [36]. Difficulties with menstruation can lead to anemia, impact the quality of life negatively and place an enormous socio-economic burden on women as well as their families, health services and society [37]. The International Federation of Gynecology and Obstetrics (FIGOs) has defined the standardized parameters for typical menstruation in terms of menstrual rate, duration, regularity and volume, and deviation from these may constitute abnormal uterine bleeding [38]. The characteristics of the menstrual cycle increasingly act both as indicators and as possible determinants of general health and well-being [39]. Abnormal uterine bleeding, amenorrhea, dysmenorrhea, premature menopause (primary ovarian insufficiency) and premenstrual syndrome are the main menstrual disorders reported. To these we can add those which are reproductive organ-dependent but not specific to the menstrual cycle, such as pelvic congestion syndrome and polycystic ovaries [40].

**Figure 1:**
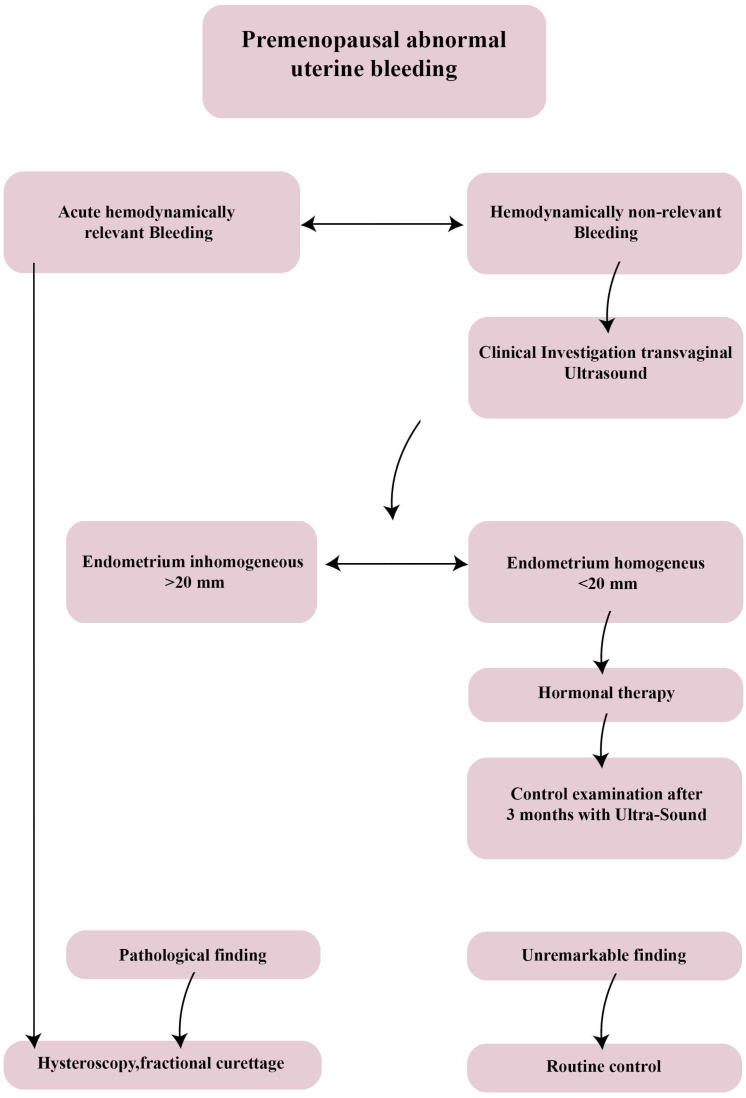
Diagnostic algorithm for abnormal premenopausal uterine bleeding. In the clinical work-up, a hemodynamically significant bleeding should be investigated directly with a II level exam/hysteroscopy, while a leakage of blood of inferior amount is generally evaluated in first instance by a transvaginal ultrasonography.

Furthermore, long and irregular menstrual cycles have been linked with a higher risk of premature mortality, and rare or absent menstruation may indicate the possibility of reduced fertility and a risk of a number of chronic conditions [40]. Menstrual bleeding (Figure 1) and the severity of (pre) menstrual symptoms are influenced by the complex interaction between hormones and the immune, vascular and coagulant systems which regulate the menstrual cycle [41].

Biologically, abnormal uterine bleeding usually occurs if estrogen levels are elevated rather than decreased, as is the case with a released but unfertilized egg. This event, not balanced by an adequate level of progesterone, causes a thickening of the endometrium (also called endometrial hyperplasia) contrary to its physiological flaking. The result is therefore a condition in which, periodically, the epithelium flakes off incompletely and abnormally, causing equally irregular bleeding [41]. These changes occur as a result of effects on hypothalamic–pituitary–ovarian–endometrial function and it is plausible that effects at the hypothalamus, pituitary or ovarian–endometrial level may cause such alterations, respectively. When abnormal uterine bleeding in pre-menopause occurs, particularly if this involves a hemodynamic decompensation, it is necessary to provide immediate hospitalization to perform an urgent fractional curettage or hysteroscopy, following stabilization of the circulation and evaluation of the blood count [42].

On the contrary, in the case of abnormal uterine bleeding with stable hemodynamics, it is possible to conduct outpatient management with routine diagnostic tests, such as ultrasound. This makes it possible to distinguish patients with endometrial thickening less than 20 mm of homogeneous septum, for which the patient can be referred to medical therapy and re-evaluation at three months [42]. After that, if the endometrial aspect is pathological, a further diagnostic investigation with hysteroscopy should be recommended, and otherwise only follow-up [42]. On the other hand, in the case of an endometrium greater than 20 mm and with uneven appearance, a level II diagnostic assessment such as hysteroscopy must be performed to exclude dysplastic degeneration of the endometrium [41]. In fact, abnormal thickening and irregular cleavage can cause the development of precancerous cells and endometrial cancer, even in young women [42].

## 3. Alterations of the Menstrual Cycle as a Result of COVID-19 Infection

**Figure 2:**
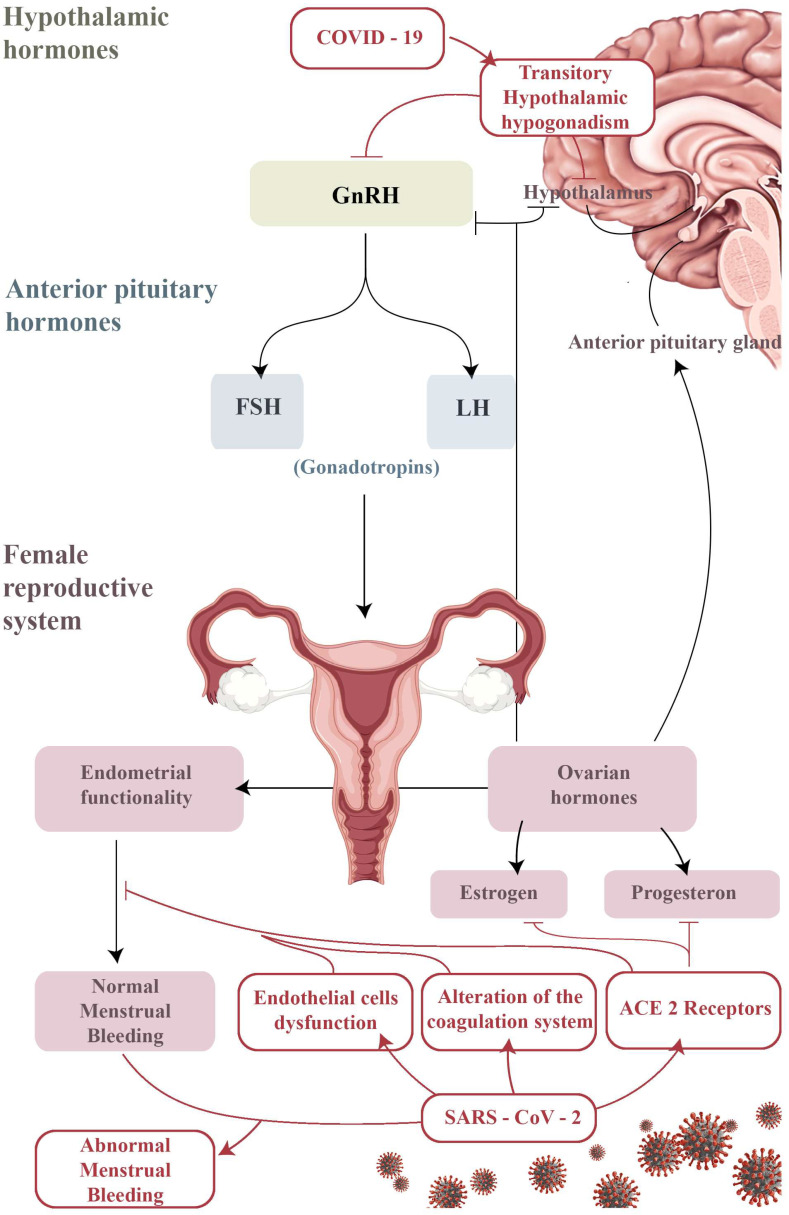
The hypothalamic–pituitary axis ovary endometrium; a summary table of the possible correlations between SARS-CoV-2 infection and transitory alterations of the neuroendocrine and gynecological systems. COVID-19 may lead to a transient suppression of the physiological secretory pulsating activity of the hypothalamic Gonadotropin-Releasing Hormone (GnRH), thus resulting in a temporary hypothalamic hypogonadism. Moreover, SARS-CoV-2 binding to ACE-2 receptors, assumed to be present in endometrial and ovarian tissue as well, could bring to alterations in endocrine pathways and/or coagulation disorders, in both cases resulting in abnormal menstrual bleeding.

After more than two years of pandemic, there is evidence of more women infected with COVID-19 experiencing changes in their menstrual cycle [34]. As already reported, the menstrual cycle generally consists of complex interactions of the hypothalamus, pituitary, ovaries, uterus, prostaglandins and neuroendocrine factors [43], as well as menstrual disorders that can be caused by a disruption of any of these interactions [3,44]. Hypothalamic hypogonadism can occur in the presence of any serious disease, including COVID-19, and cause temporary amenorrhea or infrequent menstruation [45]. This protective mechanism allows for energy resources to be diverted from reproduction to immune response [46]. This could also explain the menstrual cessation or irregularity reported amongst women experiencing long-term symptoms of Ebola infection (post-Ebola syndrome; possibly analogous to long COVID-19) [47]. Alternatively, or in addition, at the ovarian/endometrial level there could be more specific interactions between the reproductive system and SARS-CoV-2 infection. The ovarian hormone progesterone is predominantly anti-inflammatory [48]. Levels of progesterone drop dramatically before menstruation, causing an influx of inflammatory cells to the endometrial environment. This leads to the shedding of the functional endometrium during menstruation [49]. Menstrual blood loss is limited by an intense vasoconstriction of specialized endometrial spiral arterioles and activation of the local coagulation system. It is proposed that ACE2 receptors are present on ovarian and endometrial tissue [50] and therefore it is hypothesized that SARS-CoV-2 infection could affect ovarian hormone production as well as endometrial response to menstruation (Figure 2).

For example, the altered number/phenotype of endometrial leukocytes during or after SARS-CoV-2 infection can potentially influence menstrual blood loss. Previous research shows that immune disruption induced by viral infection is associated with an exacerbation of progesterone-related premenstrual symptoms [51]. In addition, COVID-19 has also been linked with two critical compounds of endometrial function at menstruation, endothelial cell dysfunction and alterations in the coagulation system, which potentially indicates an endometrial mechanism for menstrual disorder [52]. Reciprocally, the severity of COVID-19 can also be influenced by reproductive hormones and/or menstruation, and the severity of COVID-19 symptoms can differ at different stages of the menstrual cycle [53]. In a recent study by Davis et al. looking at long COVID-19 symptoms, more than a third of the participants experienced relapses of symptoms during or before menstruation, i.e., during the most inflammatory phases of the cycle [54]. Cyclic variations in symptoms have also been reported in those with myalgic encephalomyelitis/chronic fatigue syndrome (ME/CFS), a chronic condition often triggered by infection which has been compared with long COVID-19. Female ME/CFS patients often report flare-ups of their symptoms during the premenstrual phase of their cycles or at the start of menopause [3]. Other studies have also reported changes in the menstrual cycle after COVID-19 infection, either in terms of the number of days between two cycles or as regards length of menstruation or amount of blood lost. In normal circumstances, these variations in the menstrual cycle can be influenced by psychological disorders (especially stress and depression) [53], well documented as long-term symptoms of COVID-19 infection [55]. In a previous study conducted in the United States, it was found that COVID-19-related stressors may be a contributing factor to changes in the menstrual cycle. In that study, women displayed a high level of perceived stress during the COVID-19 pandemic, and in the same period reported significant changes in their menstrual bleeding [54].

## 4. Alterations of the Menstrual Cycle after COVID-19 Vaccination

**Figure 3:**
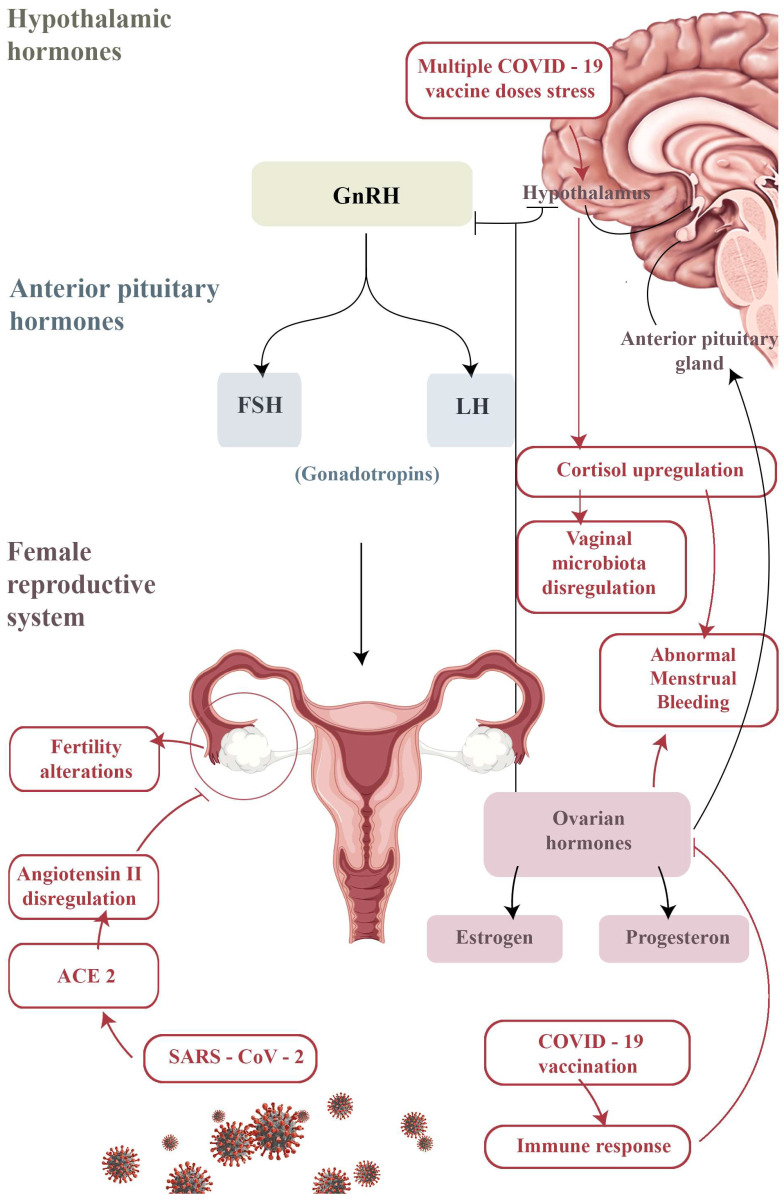
Schematic representation of the possible mechanisms underlying the neuroendocrine and gynecological adverse reactions to the SARS-CoV-2 vaccine. As in the case of the COVID-19 effect, acute stress induced by multiple vaccine doses could temporarily impair the hypothalamic–pituitary–gonadal axis by interfering with pulsatile GnRH secretion; otherwise, an upregulation of cortisol mediated by abnormal secretion of cortisol-releasing hormone (CRH) could lead to a dysregulation of the vaginal microbiota and ultimately to abnormal menstrual bleeding. An excessive immune response to the vaccine could also trigger a blockage of the aromatase enzyme, thus causing the same symptoms.

To date, administered vaccines include the Pfizer/BioNTech mRNA vaccine Comirnaty (43%), the mRNA Moderna vaccine known as COVID-19 Modern vaccine (32%) and the recombinant viral vector vaccine AstraZeneca, now named Vaxzevria (25%) [56]. Most reports concern the Pfizer/BioNTech Comirnaty vaccine (75%), which was the most used (70.9% of doses administered), whereas only a small number reference the Vaxzevria (formerly AstraZeneca COVID-19; 22%) or the Moderna vaccine (3%) [24]. In addition, adverse effects after the vaccine appear to be numerically higher in direct proportion to the number of doses [24]. For these administered vaccines, AIFA and the Medicines and Healthcare Products Regulatory Agency (MHRA) have reported fever, fatigue, headache, muscle/joint pain, injection site pain, chills and nausea as adverse events [56]. Gathering as much information as possible on all events that occur after vaccination is necessary in order to increase the possibility of identifying suspected events [24]. However, a causal link with vaccination is not always easy to assess [32], as events could be for example a symptom of a different disease or be caused by another product taken by the vaccinated person [57]. It should be noted that the reports of adverse events by AIFA represent only a small percentage of the reports present in the National Pharmacovigilance Network at the time of data extraction and could change in time [24]. In gynecological clinical experience, an increasing number of patients have reported alterations of the transient period and menstrual irregularities shortly after vaccination [56]. Similarly, since 2 September 2021, at the MHRA the literature has reported these types of reactions across all COVID-19 vaccines [58]. Previous analysis of reports of post-vaccine menstrual disorders by the MHRA had concluded that there was no causal link between alterations in the menstrual cycle and vaccines [59]. In view of further reports on menstrual disorders, however, they decided to investigate the incidence of such cases and to carry out a re-analysis of all available data, which is currently ongoing [56]. In addition, any changes in the menstrual cycle after vaccination seem to return to normal by the next cycle [60]. The mechanism of these adverse reactions has not yet been sufficiently explored. For this reason, the United States National Institutes of Health are investing many resources in trials, which are already underway with case–control groups (vaccinated and unvaccinated). Awaiting the results of these ongoing studies, we carried out a retrospective survey of the vaccinated population of our outpatient services to assess, over a period of one year, whether vaccinated patients had reported adverse reactions. Several questions arose about the impact of SARS-CoV-2 infection and vaccination on future fertility [61]. In particular, with regard to male fertility, the literature reports an absence of SARS-CoV-2 in the semen and prostatic secretions of infected patients [1,61]. In patients with a recent infection or those recovering from COVID-19, the possibility of sexual transmission through sperm approximately 1 month after the first diagnosis is unlikely [1], and SARS-CoV-2 RNA was not detected in sperm either during the period immediately following infection or subsequently [2]. There are indirect viral signs, such as testicular lesions and inflammatory infiltration, viral orchitis, scrotal discomfort and altered sperm parameters (such as sperm count with DNA fragmentation). SARS-CoV-2 can lead to sterility through the main receptor which binds the ACE2 E2 receptor, widely distributed in the testes, including Leydig and Sertoli cells [60]. Further studies are needed to investigate these aspects and the impact of “long COVID-19” on male reproduction [32]. On the other hand, COVID-19 vaccination does not appear to harm the sperm quality and fertilization ability of men (especially undergoing ART treatments), regardless of the type (mRNA or viral vector), and should therefore be considered safe for men’s reproductive health [62]. Conversely, for females, SARS-CoV-2 can invade target ovarian cells by binding to ACE2, which is widely expressed in the uterus, ovaries, vagina and placenta. It regulates angiotensin II levels to exert its physiological functions, which include follicular development and ovulation, angiogenesis and corpus luteum degeneration, as well as affects endometrial tissue growth. Ovarian reserve function should therefore be assessed in order to analyze the impact of COVID-19 on female fertility [57]. Despite a previous low incidence of severe morbidity among pregnancies affected by COVID-19, recent publications have reported both severe morbidity and mortality among pregnant women affected by emerging variants of the SARS-CoV-2 virus. As a result, the Center for Disease Control added pregnancy to the list of high-risk conditions to prioritize vaccination, and the American College of Obstetricians and Gynecologists now recommends vaccination regardless of pregnancy stage [63]. Regarding women wanting offspring, COVID-19 vaccination does not appear to have an impact on fertility, with clinical trials reporting similar rates of adverse pregnancy outcomes within vaccinated and unvaccinated cohorts [64]. Similarly, fertility measures and pregnancy rates in assisted reproduction clinics are similar in vaccinated and unvaccinated patients [65]. Many women have reported menstrual disorders after COVID-19 vaccination, including alterations in the regularity, frequency, volume and duration of menstruation [66]. It is not easy to understand whether these disorders are directly caused by the vaccine itself, or understand the mechanisms that cause these effects, since this can vary from person to person [67]. In fact, considering that the female system is designed to temporarily reduce regulation to prevent pregnancy and conserve energy at stressful times, changes in menstruation could simply be due to stress [68]. This mechanism could account for some of the menstrual irregularities detected during the COVID-19 pandemic [69]. On the other hand, COVID-19 vaccination gives rise to an immune response, and the subsequent inflammation can transiently disrupt ovarian hormone production for one or two cycles, resulting in abnormal menstrual bleeding MHRA (Figure 3) [70].

Regarding this hypothesis, a recent study evaluated the ovarian involvement in the immune reaction to COVID-19 vaccination [71]. This research revealed the presence of anti-SARS-CoV-2 IgG in serum and follicular fluid in recently vaccinated patients compared to uninfected unvaccinated women who were candidates for IVF [72]. The research showed that follicular steroidogenesis displayed similar, normal rates of estrogen and progesterone production between groups. An evaluation of the follicular response to the LH/hCG trigger also showed a normal and similar response in the different groups. Therefore, there were no measurable changes in oocyte maturation and the hormonal environment compared to unexposed patients, despite the evidence of close follicular immune exposure post-infection with SARS-CoV-2 or after BNT162b2 mRNA vaccine [73]. Another suggestive hypothesis predicts that following the stress of multiple COVID-19 vaccine doses, the hypothalamus–pituitary–adrenal axis tends to exacerbate the stimuli related to the production of cortisol (Figure 3), which notoriously affects the female reproductive tract or can even modify the vaginal microbiota [74]. This has also been established in other endometrial pathologies [67]. Our study [12] is a pilot experience and is limited by the fact that it is retrospective, but it could represent a milestone for future studies. In particular, the observation period and sample size should be extended with a multicenter study in order to gather more reports and evidence and implement the AIFA’s acquisitions. In fact, due to a lack of an evident causal link between disorder and COVID-19 vaccine, the number of official reports is currently small, both compared to the number of vaccinated people and the general incidence of menstrual disorders [75]. A real understanding of the mechanism of a hypothetical association between COVID-19, COVID-19 vaccines and menstrual changes can only be reached through the implementation of this research. However, it is important to emphasize, especially for women who would like offspring, that according to today’s knowledge the effects on menstrual symptoms are not cause for concern since they are transient, resolve spontaneously and are much less severe than those associated with COVID-19 infection. Therefore, both COVID-19 positive patients and patients who have recently undergone vaccination must be informed of the possibility of having transient alterations to their menstrual cycle, but advised that when these persist further specialist investigations may be required.

## 5. Conclusions

As the world begins to recover from the COVID-19 pandemic, more research is needed to help us to understand and mitigate its impact on menstrual health, potentially also helping to minimize gender-based health and social inequalities. The pandemic has highlighted the need for further research to reach a greater understanding of how external environmental factors can affect the menstrual cycle and how it can influence other health aspects in a bidirectional way. It will be necessary for researchers to consider whether they can unravel and identify the effects of various factors (pandemic mitigation/control factors, acute COVID-19, long COVID-19, treatments, vaccines) for an increasing percentage of the population that has been exposed to most if not all of them. Future studies should adequately consider and describe the situation with the pandemic (e.g., type of restrictions, their duration, how they were enforced, level of compliance, etc.), social attitudes toward menstruation, menstrual health awareness as well as availability and accessibility of menstrual products and health services for their target populations. These factors provide the much-needed social context which will allow for the results to be interpreted and compared across different populations. However, it is possible that existing research cohorts, digital fitness trackers and smartphone apps tracking menstrual cycles may have already picked up some of these repeated measures. Each of these methods has its pros and cons. For example, data from smartphone menstrual cycle tracking apps are collected on a large number of women frequently and longitudinally, but the data are influenced by a high degree of deficiency and collected from only a select group of smartphone users, many of whom use the app to track fertility as they try to become pregnant. Collecting relevant data on menstrual cycle changes within existing cohort studies would also allow for longitudinal data collection, and the lack may be lesser (and potential selection bias can be studied with existing data), but the frequency of repeated data collection and sample size are likely to be lower than in the datasets collected by smartphone apps. Cautious interpretations from individual studies will be required. Triangulating the evidence gathered using different approaches with different and unrelated key sources of bias may allow us to draw more conclusive inferences about the nature of any causal effect. Exploring the heterogeneity between studies would help us understand both how the context may moderate the effect of various exposures and also the extent to which results could be biased. We ask anyone with such data to contact the authors to discuss the possibility of setting up such a network. Anecdotal evidence discussed online, government monitoring systems and a small number of scientific studies of varying quality suggest that many women have experienced changes in the characteristics of their menstrual cycle during the COVID-19 pandemic, due to pandemic-related factors such as stress and behavioral changes and/or due to the COVID-19 disease itself.

In conclusion, the evidence currently at our disposal highlights that COVID-19 effects tend to have a transitory effect on the female reproductive system and are “GONE WITH THE WIND”. This condition, however, deserves further investigation both to verify whether this transience is the cause of further effects and to monitor whether future COVID-19 variants do not exclude long-term symptoms.

## Figures and Tables

**Table 1:** Studies that report the characteristics of the menstrual cycle during the period of the COVID-19 pandemic.

ID	Year	Authors	Title	Study Design	Country	Age (Mean Years Range)	Sample Size
[28]	2022	Buran and Gercek	Impact of the awareness and fear of COVID-19 on menstrual symptoms in women: A cross-sectional study	Cross-sectional	Turkey	27.1, 18–42	125
[29]	2021	Nguyen et al.	Detecting variations in ovulation and menstruation during the COVID-19 pandemic, using real-world mobile app data	Cohort	Great Britain, United States, Sweden, other countries	32.5, N/A	18,076
[30]	2022	Ozimek et al.	Impact of stress on menstrual cyclicity during the coronavirus disease 2019 pandemic: A survey study	Cohort	United States	32.5, 18–45	210
[31]	2021	Phelan et al.	The impact of the COVID-19 pandemic on women’s reproductive health	Cross-sectional	Ireland	36.7, 15–54	1031
[32]	2021	Takmaz et al.	The impact of COVID-19-related mental health issues on menstrual cycle characteristics of female healthcare providers	Cross-sectional	Turkey	29.5, 18–40	952
[33]	2022	Maher et al.	Female reproductive health disturbance experienced during the COVID-19 pandemic correlates with mental health disturbance and sleep quality	Cross-sectional	Ireland	N/A	1335
						29–38	

**Table 2:** Studies that report the characteristics of the menstrual cycle in relation to the COVID-19 vaccination.

ID	Year	Authors	Title	Study Design	Country	Age (Mean Years Range)	Sample Size
[34]	2022	Edelman et al.	Association Between Menstrual Cycle Length and Coronavirus Disease 2019 (COVID-19) Vaccination: A U.S. Cohort	Cohort	U.S.A.	27.1, 18–45	3959
[35]	2022	Laganà et al.	Evaluation of menstrual irregularities after COVID-19 vaccination: Results of the MECOVAC survey	Survey	Italy	35.8	369
						18–45	
[12]	2022	Dellino et al.	SARS-CoV-2 Vaccines and Adverse Effects in Gynecology and Obstetrics: The First Italian Retrospective Study	Survey	Italy	32.5, 18–45	100

## Data Availability

Not applicable.
